# Induction of fertile estrus without the use of steroid hormones in seasonally anestrous Suffolk ewes

**DOI:** 10.5713/ajas.18.0769

**Published:** 2019-04-15

**Authors:** Erika Elizabeth Miguel-Cruz, Octavio Mejía-Villanueva, Luis Zarco

**Affiliations:** 1 Ovine Teaching and Research Farm, Faculty of Veterinary Medicine, National Autonomous University of Mexico, Tres Marias, Morelos 62515, Mexico

**Keywords:** Male Effect, Induction of Ovulation, Seasonal Anestrus, Suffolk Ewe, Steroid-free, Fertile Estrus

## Abstract

**Objective:**

To evaluate the efficacy of treatments based on gonadotrophin-releasing hormone (GnRH), GnRH-prostaglandin F2α (PGF2α), and/or intense exposure to novel rams to induce fertile estrus without the use of steroid hormones in seasonally anestrous Suffolk ewes.

**Methods:**

In the first experiment, ewes were treated with one injection of GnRH, two injections of GnRH administered 7 days apart, or a sequence of GnRH-PGF2α-GnRH (GPG). In the second experiment anestrous ewes were exposed, for 36 days starting on the day of weaning, to groups of four rams of three different breeds that were alternated every day. Besides exposure to the male effect (ME), the ewes were injected with saline solution (ME group, n = 20), with GnRH (ME-GnRH group, n = 20) or with a sequence of GnRH-PGF2α-GnRH (ME-GPG group, n = 20). The rams used for male-effect were fitted with aprons to prevent mating, and ewes detected in estrus were bred to selected fertile rams. Ovarian activity was monitored by progesterone determinations in both experiments.

**Results:**

In the first experiment sustained induction of ovarian activity was not achieved and no ewe was detected in estrus. In the second experiment induction of sustained ovarian activity was achieved in all groups. Most of the ewes were detected in estrus, 76.7% of the ewes were mated during a 36-d breeding period and 71.7% of all the ewes became pregnant during that period. No significant differences between groups were found for any of these variables. However, estrus detection efficiency was higher in the ME-GnRH group than in the ME group (p<0.05).

**Conclusion:**

An intense male-effect, that included the continuous presence and frequent alternation of several rams of different breeds, was sufficient to induce ovarian activity and fertile estrus in Suffolk ewes during the period of deep anestrus without the use of hormones, although addition of GnRH improved the efficiency of estrus detection.

## INTRODUCTION

Intensive lamb production systems involve the obtention of more than one lambing per ewe per year. Accelerated lambing systems require the implementation of two, three or even five breeding periods at different times of the year [1,2]. When these systems are used to manage highly seasonal ovine breeds, at least one of the breeding periods coincides with the middle of the anestrous season. Poor reproductive efficiency is attained at that time, and this affects the overall productivity of the system [1–3].

Induction of ovarian activity during seasonal anestrus is best achieved through the use of progesterone (P4) or synthetic progestogens [4], so that accelerated lambing systems often include the use of these hormones during the periods of low or absent natural ovarian activity [1,3]. However, consumer concerns regarding negative health and environmental effects from the use of steroid hormones in animal production are increasing [5,6]. These concerns have motivated the development of methods for induction of fertile ovarian activity in anestrous animals without the use of steroid hormones [5–8]. Highly seasonal breeds of sheep, such as the Suffolk ewe, represent an additional challenge, as they are less responsive than other types of sheep to the methods used to induce out-of-season ovarian activity [5].

Several protocols that combine gonadotrophin-releasing hormone (GnRH) and prostaglandin F2α (PGF2α) have been used with reasonable success in sheep to synchronize estrus and ovulation without the use of progestogens during the natural breeding season [9]. However, very little information is available regarding the use of such protocols during the anestrous season. Published studies on the use of GnRH-PGF2α protocols in anestrous ewes relied on estrus detection to assess their efficacy, and P4 determinations were not made to characterize the actual ovarian effects of the treatments [10,11]. Some of the GnRH-PGF2α combinations showed potential for steroid-free induction of ovarian activity, but the lack of P4 measurements prevents complete interpretation of the findings. Thus, the first experiment of this study aimed to characterize the ovarian response of anestrous Suffolk ewes to protocols based on GnRH or GnRH-PGF2α combinations.

Another alternative for steroid-free induction of ovarian activity without the use of hormones would be the use of appropriate male-effect protocols. Induction of fertile estrus during the anestrous season can be achieved in ewes of low-seasonality breeds by means of the male effect alone [5,12,13]. In contrast, ewes of the highly seasonal Suffolk breed have proved to be very difficult to induce into effective ovarian activity by the male effect during the middle of the anestrous season, and even during the transition into the breeding season [5,14–16]. However, Nugent and Notter [15] improved the effectivity of the ram effect in Suffolk ewes by mixing them with less seasonal white-faced ewes, which probably amplified the effect of the males by female-to-female stimulation [17]. Also, Clemente et al [18] found an improved response of Suffolk ewes to the male effect when rams of a low-seasonality breed were used for stimulation. The results of those studies suggest that the male effect can be effective in Suffolk ewes if the stimulus is intense enough. One possibility to achieve the required intensity would be to take advantage of the “male novelty” factor [13,19]. For this reason, intense alternate exposure to males of different breeds was used in the second experiment of this study.

Combining the male effect with steroid-free hormonal treatments may help to improve the results of programs of induction of ovarian-activity in highly-seasonal breeds. It has been shown in Karakul ewes that the efficacy of GnRH-PGF2α protocols to induce ovarian activity in anestrous ewes can be improved by their combination with the male effect [20]. However, Karakul is a breed with an extended breeding season, so that the combination of the male effect with GnRH-PGF2α treatments remains to be studied in highly seasonal breeds. Thus, the ultimate objective of the present study was to evaluate the efficacy of treatments based on GnRH, GnRH-PGF2α, and/or intense exposure to novel rams to induce fertile ovarian activity without the use of steroid hormones in seasonally anestrous Suffolk ewes.

## MATERIALS AND METHODS

The experiments were approved by the Institutional Committee for the Care and Use of Experimental Animals of the Faculty of Veterinary Medicine of the National Autonomous University of Mexico, according to Mexican Official Norm NOM-062-ZOO-1999 [21]. Two experiments were conducted at an experimental farm located in a temperate region of Mexico, at 19° 02’ N, 99° 16’ W. At this latitude the length of the longest day of the year is 13 h 6 min and the shortest day is 10 h 54 min [22]. During the experimental period the daylength increased by an average of 1 min and 12 s/d.

### Experiment 1

#### Animals and treatments

The experiment started during the first week of May, when Suffolk ewes in this region are in deep anestrus [22]. Thirty-two adult Suffolk ewes were used. Lack of ovarian activity before the onset of the experiment was confirmed by P4 concentrations below 0.5 ng/mL in three consecutive samples taken at five-day intervals. The age of the ewes ranged from two to five years and their weight was 83.2±4.3 kg. The animals had lambed during February and the lambs were weaned two weeks before the onset of the experiment.

**Figure 1:**
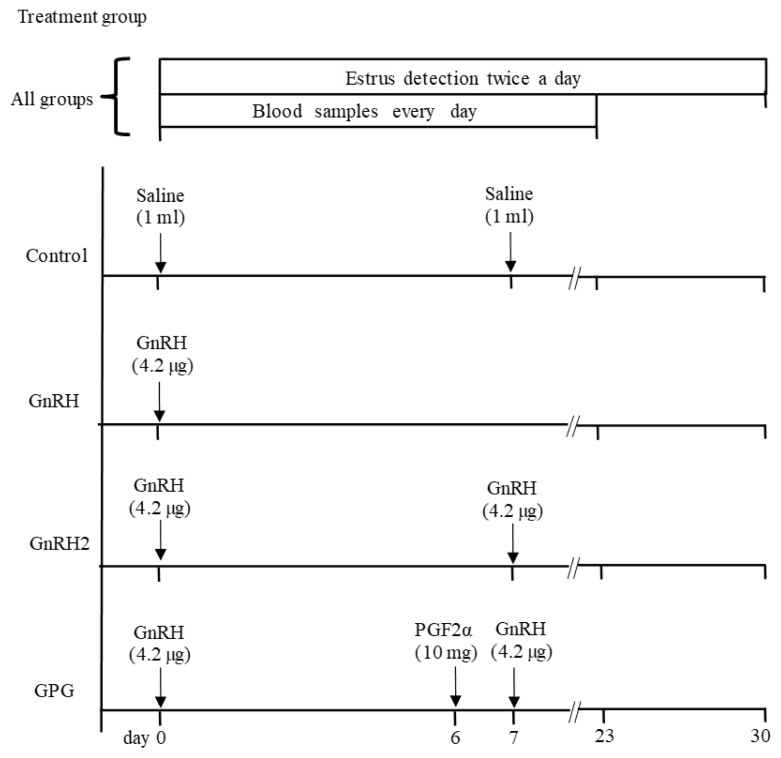
Schematic diagram of the schedule of activities and treatments in experiment 1.

A schematic representation of the schedule of experiment 1 is shown in Figure 1. The ewes were randomly divided into four groups. The control group (n = 8) was treated with 1 mL physiological saline solution on day 0. Ewes in the GnRH group (n = 8) were treated with 4.2 μg of GnRH (Buserelin acetate, Conceptal, MSD Animal Health, Mexico City, México) on day 0. Animals in the GnRH2 group (n = 8) were treated with the same dose of GnRH on days 0 and 7. Ewes in the GPG group (n = 8) received GnRH on day 0, followed by 10 mg of PGF2α (Dinoprost tromethamine, Lutalyse, Zoetis, Mexico City, México) on day 6 and a second injection of GnRH on day 7. All the ewes were kept together in a single barn and estrus detection was carried out twice a day for 20 min from day 0 to day 30 using two Dorset rams fitted with aprons to prevent mating. The active condition of the rams had been previously verified by assessing their behavior when exposed to two ewes induced into estrus with progesterone pessaries. Heparinized blood samples were collected from the jugular vein every day from day 0 to day 23. The samples were centrifuged within 20 min (1000 G for 10 min) and the plasma was separated and kept frozen until assayed using a commercial solid-phase RIA kit (Coat-A-Count P4; Siemens Medical Solutions, Los Angeles, CA, USA). The sensitivity of the assay was 0.13 ng/mL, with intra-assay and inter-assay variation coefficients of 5.5% and 8.4%, respectively.

#### Definitions

The ewes of each group were classified according to their response as: No response: when P4 concentrations never increased above 0.75 ng/mL; Short-cycle only: when P4 concentrations increased above 0.75 ng/mL in at least one sample but remained elevated less than 7 days and did not increase again; Two short cycles: when two consecutive luteal phases lasting less than 7 days each were detected; and Short+ normal cycle: when a short cycle was followed by a subsequent increase in P4 concentrations above 0.75 ng/mL that lasted for 8 or more days.

#### Statistical analysis

The proportion of no response was compared between groups by the Freeman-Halton extension of the Fisher’s exact test for 4×2 contingency tables [23] using the VassartStats statistical computation Web site.

### Experiment 2

#### Animals and treatments

The experiment was started during the third week of April. Sixty adult Suffolk ewes that had lambed in February and were weaned between day 55 and 65 post-lambing were used. The age of the ewes ranged from two to six years and their weight was 78.2±3.8 kg. Blood samples for P4 determination had been obtained every 3 days during the three weeks prior to weaning in order to verify that the animals were not cyclic. During that period the ewes remained with their lambs in collective pens.

Once weaned the ewes were moved into pasture, where they remained every day from 08:30 to 15:30 h. The rest of the day and at night they stayed in a pen, where they had free access to water and received a supplement with oat-hay, commercial concentrate and corn silage. The 60 ewes remained together throughout the experiment.

**Figure 2:**
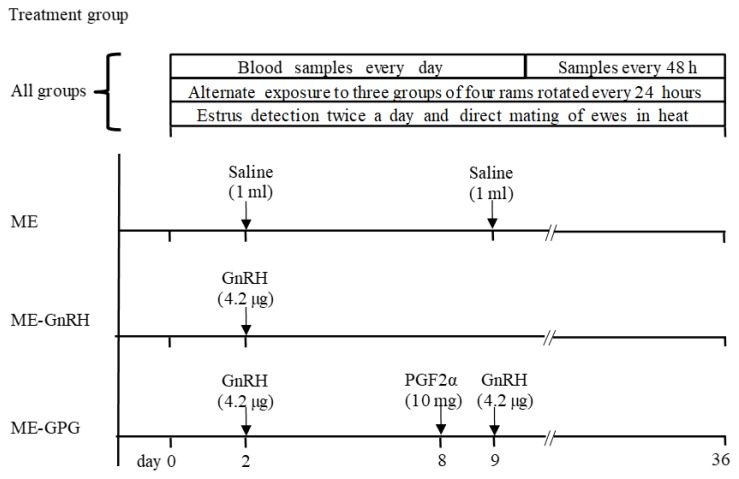
Schematic diagram of the schedule of activities and treatments in experiment 2.

A schematic representation of the schedule of experiment 2 is shown in Figure 2. Starting at the time of weaning the ewes were exposed for 36 days to the continuous presence of rams fitted with aprons to prevent mating. Twelve sexually active rams of three different breeds (Suffolk, Hampshire and Dorset) were rotated every 24 h in groups of four in order to provide for the constant presence of four males, while maintaining male-novelty. The day of weaning and of the onset of exposure to the males was considered as day 0 of the experiment. The active condition of the males had been previously assessed as described in experiment 1.

On day 2 the ewes were randomly assigned to one of three treatments. The ewes in the “male effect” group (ME, n = 20) were injected with physiological saline on days 2 and 9 and remained exposed to the males for 36 days. Ewes in the ME-GnRH group (n = 20) were exposed to the males for 36 days and treated with 4.2 μg of GnRH on day 2. Ewes in the ME-GPG group (n = 20) remained exposed to the males for 36 days and were treated with 4.2 μg of GnRH on day 2, 10 mg of PGF2α on day 8, and 4.2 μg of GnRH on day 9.

Blood samples for P4 determination were obtained once a day for the first 10 days of the experiment, and every 48 h thereafter until day 35. The samples were processed and analyzed as described in experiment 1. Detection of estrus was carried out twice a day for periods of 30 minutes while the ewes were in the pen, early in the morning and at late afternoon. Three active Dorset rams, different from those used to provide the male effect, were used for estrus detection. The teasers were also fitted with aprons to prevent mating. Ewes detected in estrus were taken to a separate pen for direct mating with the male assigned to each of them according to the genetic program of the farm. Once served, the ewes were returned to the group in order to contribute to stimulation of their herd-mates through the female-to-female effect [17], and they were returned to service with the same male every 12 h while they continued to be in estrus. All the rams used for breeding were of proven fertility, in breeding condition and with good-quality semen. Pregnancy was diagnosed by ultrasound at day 40 post-service. Prolificacy was determined at the time of lambing.

#### Definitions

The day when P4 concentrations first increased above 0.75 ng/mL after the onset of exposure to the males was considered as the onset of the first luteal phase. The intervals to the onset of the second luteal phase, to first estrus and to pregnancy were also calculated from the onset of exposure to the males.

The duration of the first and second luteal phases were defined as the number of consecutive days that P4 remained above 0.75 ng/mL during the first or second luteal phases, respectively. A luteal phase was classified as short when its duration was 7 days or less, and as normal-length when it lasted from 8 to 14 days. Even though estrus was detected for 50 days after the beginning of exposure to the males, only the ewes that showed estrus during the first 36 days were served, so that only these ewes were considered to have shown an effective estrus.

It was assumed that any ovulation preceded by a normal-length luteal phase should be accompanied by signs of estrus [24], so that the absence of estrus at those ovulations was considered as a failure to detect estrus. The efficiency of estrus detection was thus defined as the percentage of the ovulations preceded by a normal-length luteal phase that were accompanied by signs of overt estrus. The pregnancy rate was defined as the proportion of pregnant ewes in relation to the ewes that showed effective estrus and were served in each group. The fertility rate was calculated as the proportion of pregnant animals in relation to the total number of ewes assigned to each group.

#### Statistical analysis

The proportion of short or normal-length first luteal phases, the proportion of ewes detected in estrus, the pregnancy rate and the fertility rate were compared between groups by the Freeman-Halton extension of the Fisher’s exact test for 3×2 contingency tables [23]. Intervals and durations were compared between groups by analysis of variance. The intervals were also compared by analysis of variance between the ewes that responded with a short first cycle and those responding with a normal-length first cycle in each treatment.

## RESULTS

### Experiment 1

**Figure 3:**
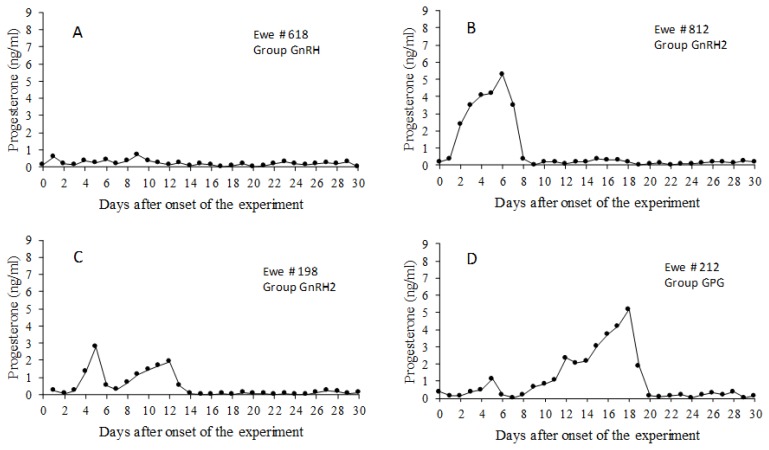
Representative progesterone profiles of the different types of response to treatment in experiment 1. (A) No ovarian response to treatment; (B) One short cycle followed by ovarian inactivity; (C) Two consecutive short cycles followed by ovarian inactivity; (D) One short cycle followed by a normal length cycle before returning to ovarian inactivity. The number of ewes with each type of response in each treatment is shown in Table 1. Treatments: GnRH: The ewes were treated with 4.2 μg of GnRH on day 0; GnRH2, Administration of 4,2 μg of GnRH on days 0 and 7; GPG, The ewes were treated with GnRH on day 0, PGF2α on day 6 and GnRH on day 7.

The results of the first experiment are summarized in Table 1. Progesterone profiles representative of the different types of response to treatments are shown in Figure 3. None of the animals in the control group showed an elevation of P4 concentrations above 0.75 ng/mL at any time. The proportion of no response was higher in this group than in the experimental groups (p<0.05). However, there were also animals without P4 elevations in each of the treated groups, accounting for 29% of all the treated animals. Another 50% of the treated animals responded with either one or two short estrous cycles before returning to ovarian inactivity. Only 21% of the treated ewes showed a normal-length luteal phase after the initial short cycle, and all of them returned to ovarian inactivity after a single normal-length luteal phase. No animal showed estrus in any of the groups during the 30-day detection period.

### Experiment 2

#### Ovarian response

**Figure 4:**
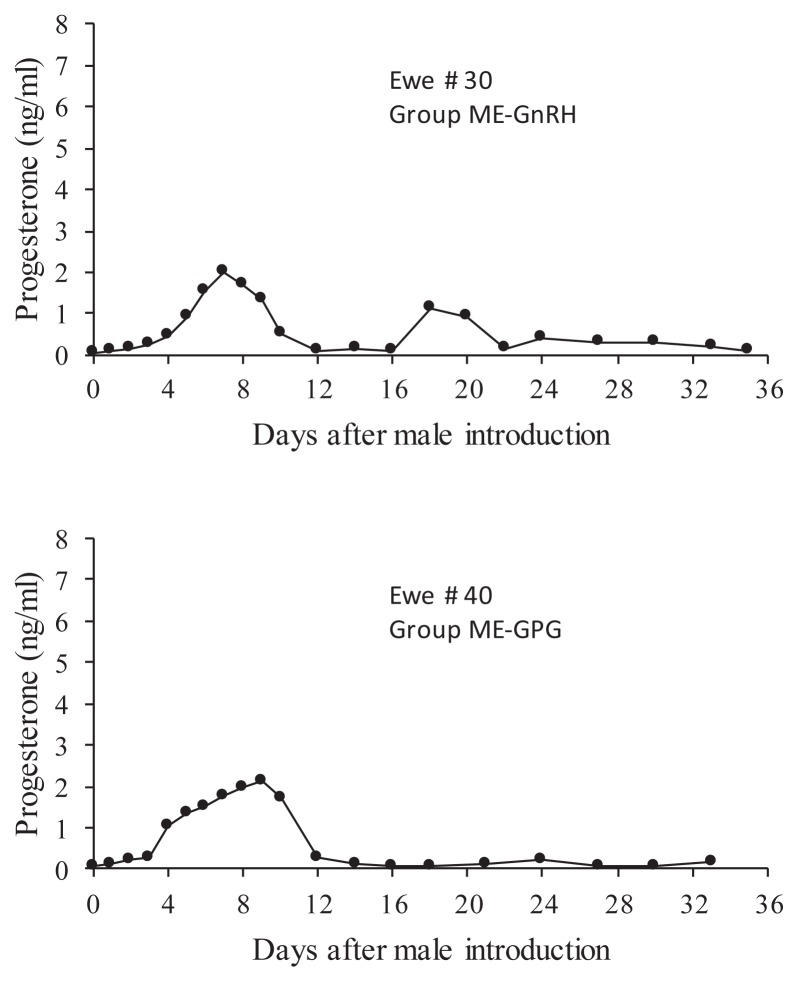
Progesterone profiles of the two ewes that returned to ovarian inactivity after one (top panel) or two (bottom panel) short estrous cycles in experiment 2.

**Figure 5:**
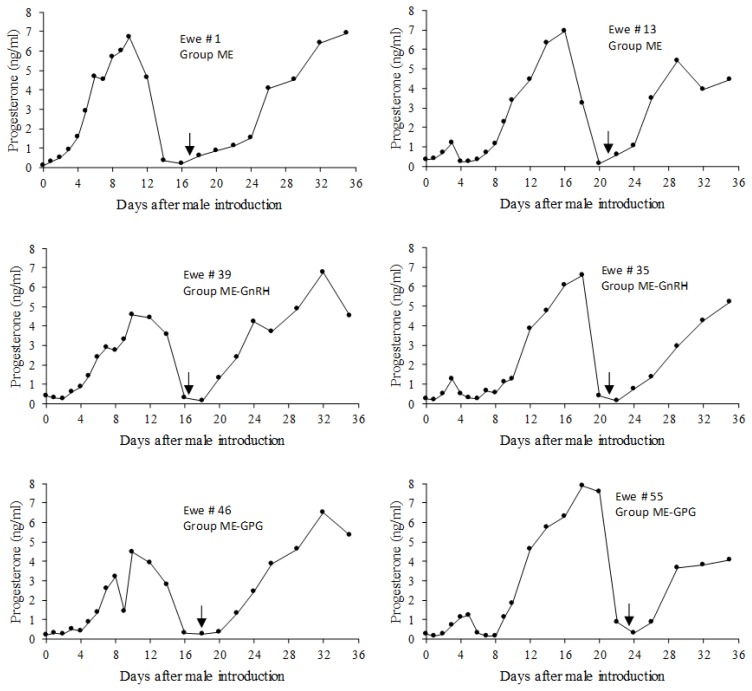
Representative progesterone profiles of ewes that responded to the treatments with a normal-length first cycle (left column) or with a short first cycle (right column) in each experimental group. Arrows indicate the onset of estrus expression. Treatments: ME, ewes were exposed from day 0 to 36 to rams of three different breeds that were rotated every 24 h in groups of 4; ME-GnRH, in addition of exposure to the males the ewes were treated with 4.2 μg of GnRH on day 2; ME-GPG, in addition of exposure to the males the ewes were treated with 4.2 μg of GnRH on day 2, 10 mg of PGF2α on day 8 and 4.2 μg of GnRH on day 9.

All the animals initiated a luteal phase within the first 7 days after the onset of exposure to the males. The first luteal phase was of short duration in 55% of the cases (Table 2). The proportion of short or normal-length first luteal phases was not different between groups (p>0.05). One animal of the ME-GPG group returned to acyclicity after one short luteal phase and one ewe in the ME-GnRH group returned to acyclicity after two short luteal phases (Figure 4). The rest of the ewes that initiated their response with a short cycle ovulated again and developed a normal-length second luteal phase. All of them, as well as all the ewes that initiated their response with a normal-length cycle, continued ovulating until they became pregnant or until P4 monitoring was discontinued on day 36. Representative progesterone profiles of ewes responding to each treatment with normal-length first cycles or with short first cycles are shown in Figure 5. Progesterone concentrations during short first cycles never increased above 2 ng/mL. In contrast, progesterone concentrations reached maximum levels between 4 and 8 ng/mL during normal-length cycles, whether or not they were preceded by a short cycle.

#### Estrus and services

There were no differences between groups in the number of ewes detected in estrus during a 50-d period, or in the number of ewes with effective estrus, i.e. those detected in estrus on time to be mated during the 36-d breeding period (Table 3). However, the efficiency of estrus detection, as defined in the material and methods section, was significantly higher in the ME-GnRH group (p<0.05) than in the ME group.

#### Reproductive efficiency

As shown in Table 4, the average pregnancy and fertility rates were 93.5% and 71.7%, respectively, without differences between groups (p>0.05). Due to the low estrus detection efficiency in the ME group, the fertility rate in this group was significantly lower than the pregnancy rate for the same group (p<0.05), and this caused the overall fertility rate for all the animals in the experiment to be significantly lower than their pregnancy rate. The total number of lambs obtained was similar in all the groups.

#### Times and intervals

Table 5 shows times and intervals for all the animals in each group, irrespective of the type of initial ovarian response of each individual. In Table 6 the same variables are shown for the ewes that responded with a short first luteal phase. Table 7 contains the information for the ewes that responded from the beginning with a normal-length luteal phase. With the exception of the ewe in the ME-GPG group that returned to ovarian inactivity after the first cycle, all the animals had at least two luteal phases, so that it was possible to calculate the interval to the first elevation of P4, the duration of the first luteal phase and the interval to the second P4 increase. In contrast, the duration of the second luteal phase could only be calculated in the animals with a short first luteal phase, since most of the animals that started their response with a normal-length cycle became pregnant immediately after the end of that cycle, so that their second luteal phase persisted until the end of pregnancy. For this reason the information of this variable is only shown in Table 6 and not in Table 5 and 7. In all the tables the value for days to first estrus includes only the animals that showed their first estrus during the 36-day breeding period.

When all the animals are considered (Table 5), there were no significant differences between groups in any of the variables related to intervals or durations (p>0.05). Within each group the interval to first estrus was the same or very similar to the interval to pregnancy because all the ewes that became pregnant did so during their first estrus. The small difference between the two variables in the group ME-GnRH is because three animals that showed estrus in this group did not became pregnant and were not included for the calculation of days to pregnancy.

In the animals with a short first luteal phase there were no significant differences between groups for any of the variables related to intervals or durations (Table 6). None of the ewes that responded initially with a short cycle showed estrus in the second ovulation. They showed estrus and became pregnant at their third ovulation, which occurred after the end of the second, normal-length luteal phase.

Among the ewes with a normal-length first luteal phase the differences between treatments were not significant for any variable (Table 7). The first luteal phase of the ewes included in Table 7 was significantly longer (p<0.05) than that of the ewes included in Table 6. Because of this, the interval to the second elevation of P4 was also longer (p<0.05) in the ewes included in Table 7 than in those in Table 6. In contrast, the ewes with a normal-length first luteal phase (Table 7) showed their first estrus earlier (p<0.05) than those with a short first-cycle (Table 6), since in the former the first estrus occurred at their second ovulation, not at their third one as in the ewes with a short first-cycle.

## DISCUSSION

In this study, none of the treatments with GnRH alone or combined with PGF2α resulted in effective induction of ovarian activity when used in the absence of the male effect. In contrasts, it was possible to induce fertile ovarian activity in a large proportion of anestrous Suffolk ewes when an intense male effect was used alone or combined with GnRH or with GnRH and PGF2α. Thus, effective induction of ovarian activity was achieved without the use of steroid hormones.

In the absence of the male effect (experiment 1), the treatments with GnRH and PGF2α did had some effect on ovarian activity, since P4 elevations were induced in 71% of the treated ewes. However, most of these animals experienced only a short cycle before returning to anestrus, and even the five animals that developed a normal-length luteal phase after their second ovulation failed to produce a subsequent ovulation. As a result, no animal showed estrus or was served in any of the groups. Thus, in contrast to what has been reported in Altamurana sheep [10] and in Corriedale ewes [11], treatments based on GnRH alone or combined with PGF2α were unable to induce fertile estrus in seasonally anestrous Suffolk ewes. Despite this, and since the results of P4 determinations provided evidence that the treatments had an initial effect on ovarian activity, we decided to evaluate the combination of these treatments with the male effect at the next anestrous season (experiment 2).

In the second experiment it was possible to induce effective ovarian activity in Suffolk ewes by continuously exposing them to rams of different breeds that were rotated every 24 h, with or without inclusion of GNRH or GnRH-PGF2α treatments. These results can be considered as very positive, since 76.7% of the ewes were served during a breeding period of just 36 days, and 71.7% of all the ewes were pregnant at the end of this limited period. The resulting lambing interval was slightly shorter than 8 months, making this a suitable method to manage ewes of highly seasonal breeds during the anestrous season in accelerated lambing systems. It is worth mentioning that the fertility rate could have been even better if the breeding period had been extended for just another week, since two ewes of the ME group showed their first estrus between day 38 and 42, and five ewes that did not become pregnant at their first estrus showed a second estrus between day 37 and 40. All these estrous periods fell outside the arbitrarily defined 36-day breeding period, but within a period that would still have allowed for an 8-month lambing interval.

There are two studies in which results comparable to those in the present study were achieved in Suffolk ewes induced to cycle by combining the male effect with a non-steroid hormone. In those studies, melatonin feeding was combined with the male effect [25,26]. Both the melatonin treatments used in those studies and the GnRH/PGF2α treatments used in this work can be considered as clean alternatives [10,25], as all these hormones degrade quickly within the animal organism, leaving no bioactive residues. However, the studies of Kusakari and Ohara [25,26] involved daily oral administration of melatonin for up to 90 days, as well as an interval of 37 to 47 days from the onset of melatonin feeding to initial exposure to the males. Thus, the first estrus in those studies occurred around day 60 after initiation of melatonin feeding, and pregnancy occurred around 76 days after the onset of the treatments [25]. In contrast, in the present study it was only necessary to administer between zero (ME group) and three (GPG group) injections to the animals, and both estrus and conception occurred on average 20 days after the onset of the experiment.

An unexpected result of the present study was the high rate of effective ovarian response observed in ewes that were only exposed to the male effect and received no other treatment, because anestrous Suffolk ewes are notoriously difficult to stimulate using the male effect without hormonal support [5,14–16]. Minton et al [16] found that only 31% of the Suffolk ewes exposed to the male effect during the period of deep anestrus (April) had a second ovulation after an initial short or normal-length luteal phase. Likewise, Nugent and Notter [15] found a complete lack of response in 34% of Suffolk ewes exposed to the males at the middle of the anestrous season (June), and only 13% of the animals responded with more than one luteal phase. Even when the rams were introduced as late as August, during the transition to the breeding season, only 40% of Suffolk ewes apparently ovulated during the first 15 days post-introduction [14]. In contrast, P4 concentrations in the present study indicate that, irrespective of the duration of their first luteal phase, all the ewes in the ME group initiated ovarian activity after exposure to the males and maintained it either until they became pregnant or until blood sampling was discontinued on day 35.

Two factors may have contributed to the good results obtained in the group that was only exposed to the male effect. The first was the intensity and variety of the stimulus, as rotational exposure to several active rams of three different breeds maintained a “novel male effect” that was frequently renovated during the study. It has been shown both in goats and in sheep that alternation of novel males during the period of exposure is more effective to induce sustained ovarian activity than permanent exposure to the same males [13,19]. The second factor was the temporal combination of the male effect with a possible stimulating effect of weaning. The ewes were exposed to the males for the first time immediately after weaning, so that both stimulating effects could have reinforced each other [27]. It would be interesting to evaluate if the male-effect protocol used in this study is effective when used in seasonally anestrous Suffolk ewes that have not been recently lactating.

We expected to improve the response to the male effect by combining it with one or two GnRH injections administered at the appropriate time to ensure ovulation of follicles previously stimulated to grow by the presence of males. The intended improvement was not evident, since an effective ovarian response occurred in the ewes exposed to the rams in all groups, irrespective of GnRH administration. However, the efficiency of estrus detection was significantly improved by the incorporation of GnRH into the induction protocol, since estrus was detected only in 60% of the ovulations that were preceded by a normal-length luteal phase in the ME group, while in the groups treated with GnRH the efficiency of estrus detection ranged from 85% to 100%. It has been reported that an important cause of failure to respond to the male effect in Suffolk ewes is the presence of silent ovulations [18,25], and that their incidence may be reduced with additional measures, such as the administration of melatonin [25] or the use of very active males of low-seasonality breeds [18]. In the present study the administration of GnRH also reduced the incidence of non-detected estrus during male-induced ovarian activity. However, it is difficult to understand the possible mechanism of action, since all GnRH administrations occurred during the first 9 days of the experiment, while the first estrus occurred on average on day 20, in association with the ovulation that occurred after the end of the first normal-length estrous cycle of each ewe. It can only be speculated that treatment with GnRH may have improved luteal function during the cycle initiated by such administration, and that additional P4 during the induced luteal phase could have facilitated expression of estrus at the onset of the following cycle.

The importance of adequate P4 concentrations during the preceding luteal phase for estrus expression is suggested, for example, by reports that estrus expression is reduced after synchronization with previously used controlled internal drug releasing (CIDRs) devices, which results in lower than normal P4 concentrations during the treatment [28,29]. However, if in the present study the differences in estrus expression between groups were related to differences in P4 concentrations during the preceding luteal phases, these differences must have been very subtle, because the general P4 profile and the average duration of the luteal phases were similar in all groups. A limitation to further evaluate this possibility is that blood samples for P4 determination were obtained only every 48 h after day 10 of the experiment, so that we do not have enough information to assess if progesterone concentrations immediately before the onset of luteolysis, or the rate of progesterone decline at the end of the normal-length luteal phases, were different between groups. Also, we have not enough data to assess if there were differences in these variables between the ewes that had silent ovulations and those that did not.

Although there is a previous report in which GnRH and GnRH+PGF2α were used to improve the response to the male effect [30], the experimental groups in that study included mixtures of animals from the highly-seasonal Suffolk breed, the low-seasonality Kathadin breed, and the intermediate-seasonality Dorset breed [30]. In that report it was concluded that GnRH may in some cases be an appropriate alternative to priming with P4 before male introduction. However, the results were not reported separately for each breed, so that the specific response of Suffolk ewes is not known. Besides, the experiments started in June, close to the natural breeding season, and the authors acknowledged that the results could probably not be applicable to the deep-anestrous period [30]. The results of the present study call into question the effectiveness of PGF2α administration a few days after induction of ovulation with GnRH. It has been reported that PGF2α can be luteolytic in the ewe as soon as day 3 post-ovulation [4], and that it has been effective when administered 5 days after the induction of ovulation with GnRH [9–11]. However, in the present study the inclusion of PGF2α 6 days after administration of the first dose of GnRH in the GPG group was ineffective to induce luteolysis in a significant proportion of animals, since 35% of the ewes in this group had a normal-length first luteal phase that was not interrupted by PGF2α administration. As exemplified by the progesterone profiles of ewe # 46, shown in the bottom-left panel of Figure 5, continuation of the luteal phase after the administration of PGF2α on day 8 of the experiment was associated with the occurrence of partial luteolysis, which has been described as the recovery of luteal function after a transient progesterone decline induced by PGF2α administration [31].

In another study, performed on low-seasonality Karakul ewes [20], the male effect was combined with GnRH. In that report it was concluded that the inclusion of GnRH as part of the induction protocol reduced the fertility of the ewes. In this regard, it is interesting that in the present study the lowest pregnancy rate in relation to the animals that were bred was attained in the ME-GnRH group. Although this reduction was not significant in relation to the other groups, it did produce a negative compensatory effect whereby the significantly better estrus detection efficiency in the ME-GnRH group did not led to more pregnancies or to more lambs produced in that group.

Although the inclusion of GnRH in the present study improved the efficiency of estrus detection, it apparently did not modify the ovarian response as such, because all the groups showed patterns of ovarian activity that were similar to those that are normally reported when ewes of less-seasonal breeds are exposed to the male effect [12,24,27]. These usual patterns include the occurrence of a short first luteal phase in a proportion of animals [24], and the expression of the first estrus only after the end of the first normal-length luteal phase, i.e. 18 to 20 days after initial exposure to the males first estrus occurred in the ewes with a first luteal phase of normal length [24] or a few days later in ewes with a short first luteal phase [24,27]. Besides, the durations of the initial short or normal-length luteal phases occurring in the different groups in this study were similar to those reported previously in ewes subjected to the male effect [24]. The absence of estrus expression during the ovulation that follows a short first luteal phase has been commonly reported in studies concerning the male effect, and it has been attributed to insufficient progesterone priming during the short luteal phase [3,12,24].

It is concluded that it is possible to induce ovarian activity without the use of steroid hormones in Suffolk ewes during the period of deep anestrus by subjecting them to an intense male-effect, with continuous presence and frequent alternation of several rams of different breeds. The main constraint in the present study was the efficiency of estrus detection, and not the ovarian response or the conception rate of the ewes. Although the inclusion of GnRH as part of the induction protocol improved the efficiency of estrus detection, it did not improve the reproductive performance of the ewes.

## Figures and Tables

**Table 1:** Classification of ovarian responses after administration of different treatments to anestrous Suffolk ewes in the first experiment

Type of response	Treatment1)			

	Control (n = 8)	GnRH (n = 8)	GnRH2 (n = 8)	GPG (n = 8)
No response2)	8^a^	3^b^	2^b^	2^b^
Short cycle only3)	0	3	5	2
Two consecutive short cycles4)	0	1	1	0
Short cycle+normal cycle5)	0	1	0	4

GnRH, gonadotropin-releasing hormone; GPG, GnRH-PGF2α-GnRH; PGF2α, prostaglandin F2α.

1) Treatments: Control, no hormonal treatment; GnRH, treated with 4.2 μg of GnRH on day 0; GnRH2, treated with GnRH on days 0 and 7; GPG, treated with GnRH on days 0 and 7, and with 10 mg of PGF2α on day 6.

2) P4 concentrations never increased above 0.75 ng/mL.

3) P4 concentrations increased above 0.75 ng/mL for 1 to 7 days.

4) Two consecutive elevations of P4 lasting 7 days or less each.

5) P4 concentrations above 0.75 ng/mL for 8 or more days after an initial short cycle.

a,b Different letters indicate differences in the proportion of ewes with no response (p<0.05).

**Table 2:** Classification of the anestrous Suffolk ewes of the second experiment according to the length of the first luteal phase after they were exposed to different treatments

Length of first luteal phase	Treatment1)			

	ME (n = 20)	ME-GnRH (n = 20)	ME-GPG (n = 20)	Total (n = 60)
Short (7 days or less)	11 (55%)	9 (45%)	13 (65%)	33 (55%)
Normal (8 or more days)	9 (45%)	11 (55%)	7 (35%)	27 (45%)

ME, male effect; GnRH, gonadotrophin releasing hormone; GPG, GnRH-PGF2α-GnRH; PGF2α, prostaglandin F2α.

1) Treatments: ME, ewes were exposed from day 0 to 36 to rams of three different breeds that were rotated every 24 h in groups of 4; ME-GnRH, in addition of exposure to the males the ewes were treated with 4.2 μg of GnRH on day 2; ME-GPG, in addition of exposure to the males the ewes were treated with GnRH on day 2, PGF2α on day 8 and GnRH on day 9.

The distribution of responses is not different between groups (p>0.05).

**Table 3:** Percent of ewes detected in estrus, percent of ewes served during the 36-d breeding period, and efficiency of estrus detection after anestrous Suffolk ewes were exposed to different treatments in the second experiment

Variable	Treatment1)			

	ME	ME-GnRH	ME-GPG	Total
Ewes detected in estrus2)	75%^a^ (15/20)	95%^a^ (19/20)	75%^a^ (15/20)	81.2% (49/60)
Ewes with effective estrus3)	65%^a^ (13/20)	90%^a^ (18/20)	75%^a^ (15/20)	76.7% (46/60)
Efficiency of estrus detection4)	60.0%^a^ (15/25)	100%^b^ (19/19)	86.3%^ab^ (19/22)	80.3% (53/66)

ME, male effect; GnRH, gonadotrophin releasing hormone; GPG, GnRH-PGF2α-GnRH; PGF2α, prostaglandin F2α.

1) Treatments: ME, ewes were exposed from day 0 to 36 to rams of three different breeds that were rotated every 24 h in groups of 4; ME-GnRH, in addition of exposure to the males the ewes were treated with 4.2 μg of GnRH on day 2; ME-GPG, in addition of exposure to the males the ewes were treated with GnRH on day 2, PGF2α on day 8 and GnRH on day 9.

2) Ewes detected in estrus at least once between days 0 and 50.

3) Ewes detected in estrus and mated during the 36-d breeding period

4) Number of estrus periods detected/number of potential estrus periods (ovulations preceded by a normal-length luteal phase) between day 0 and 50.

a,b For a given variable (line), values that do not share at least one letter are different (p<0.05).

**Table 4:** Pregnancy rate, fertility rate, prolificacy, and number of lambs produced by anestrous Suffolk ewes exposed to different treatments in experiment 2

Variable	Treatment1)			

	ME	ME-GnRH	ME-GPG	Total
Pregnancy rate (Pregnant/served)	100% (13/13)	83.3% (15/18)	100% (15/15)	93.5% (43/46)
Fertility rate (Pregnant/total)	65% * (13/20)	75% (15/20)	75% (15/20)	71.7% * (43/60)
Prolificacy	1.38	1.07	1.20	1.22
Lambs produced	18	16	18	52

ME, male effect; GnRH, gonadotrophin releasing hormone; GPG, GnRH-PGF2α-GnRH; PGF2α, prostaglandin F2α.

1) Treatments: ME, ewes were exposed from day 0 to 36 to rams of three different breeds that were rotated every 24 h in groups of 4; ME-GnRH, in addition of exposure to the males the ewes were treated with 4.2 μg of GnRH on day 2; ME-GPG, in addition of exposure to the males the ewes were treated with GnRH on day 2, PGF2α on day 8 and GnRH on day 9.

There were no significant differences between groups for any variable (p>0.5).

* Fertility rates marked with an asterisk are lower than the pregnancy rates for the same group (p<0.05).

**Table 5:** Intervals to the first and second elevations of progesterone (P4), duration of the first luteal phase, intervals to estrus and to gestation, and lambing interval for the ewes of each group, irrespective of the type of initial response to treatments

Variable	Treatment1)			

	ME	ME-GnRH	ME-GPG	Total
Days to first P4 elevation	3.5±0.5 (n = 20)	4.1±0.5 (n = 20)	3.0±0.5 (n = 20)	3.5±0.3 (n = 60)
Duration of first luteal phase	6.1±0.5 (n = 20)	5.7±0.5 (n = 20)	5.3±0.5 (n = 20)	5.7±0.6 (n = 60)
Days to second P4 elevation	15.4±1.6 (n =20)	14.6±1.6 (n =20)	12.7±1.3 (n =19)	14.2±0.9 (n =59)
Days to first estrus	20±1.7 (n = 13)	20.2±1.4 (n = 18)	19.6±1.2 (n = 15)	19.9±0.8 (n = 46)
Days to pregnancy	20±1.7 (n = 13)	19.5±1.4 (n = 15)	19.6±1.2 (n = 15)	19.7±0.9 (n = 43)
Lambing interval	223.3±2.4 (n = 13)	219.5±1.7 (n = 15)	223.2±1.8 (n = 15)	221.9±1.1 (n = 43)

ME, male effect; GnRH, gonadotrophin releasing hormone; GPG, GnRH-PGF2α-GnRH; PGF2α, prostaglandin F2α.

1) Treatments: ME, ewes were exposed from day 0 to 36 to rams of three different breeds that were rotated every 24 h in groups of 4; ME-GnRH, in addition of exposure to the males the ewes were treated with 4.2 μg of GnRH on day 2; ME-GPG, in addition of exposure to the males the ewes were treated with GnRH on day 2, PGF2α on day 8 and GnRH on day 9.

Differences between groups are not significant for any variable (p>0.05). Values are mean±standard error of the mean. Values in parenthesis indicate the number of animals that contributed to each variable.

**Table 6:** Intervals to the first and second elevations of progesterone (P4), duration of the first luteal phase, intervals to estrus and to gestation, and lambing interval for the ewes of each group that initiated their response to treatments with a short cycle

Variable	Treatment1)			

	ME	ME-GnRH	ME-GPG	Total
Days to first P4 elevation	2.7±0.7 (n = 9)	3.4±0.6 (n = 11)	3.0±0.6 (n = 13)	3.0±0.4 (n = 33)
Duration of first luteal phase	1.3±0.7 (n = 9)	1.8±0.6 (n = 11)	2.0±0.6 (n = 13)	1.7±0.4 (n = 33)
Days to second P4 elevation	8.4±1.4 (n =9)	10.4±1.3 (n =11)	9.1±1.2 (n =12)	9.3±0.8 (n =32)
Duration of second luteal phase	10.2±0.8 (n = 9)	7.8±0.7 (n = 11)	9.1±0.7 (n = 12)	9.0±0.4 (n = 32)
Days to first estrus	20.2±2.4 (n = 5)	22.4±2.2 (n = 9)	20.9±1.6 (n = 11)	21.3±1.3 (n = 25)
Days to pregnancy	20.2±2.4 (n = 5)	22.4±2.2 (n = 7)	20.9±1.6 (n = 11)	21.2±1.2 (n = 23)
Lambing interval	225.8±2.8 (n = 5)	222.5±2.6 (n = 7)	224.1±2.3 (n = 11)	224.0±1.1 (n = 23)

ME, male effect; GnRH, gonadotrophin releasing hormone; GPG, GnRH-PGF2α-GnRH; PGF2α, prostaglandin F2α.

1) Treatments: ME, ewes were exposed from day 0 to 36 to rams of three different breeds that were rotated every 24 h in groups of 4; ME-GnRH, in addition of exposure to the males the ewes were treated with 4.2 μg of GnRH on day 2; ME-GPG, in addition of exposure to the males the ewes were treated with GnRH on day 2, PGF2α on day 8 and GnRH on day 9.

Differences between groups are not significant for any variable (p>0.05). Values are mean±standard error of the mean. Values in parenthesis indicate the number of animals that contributed to each variable.

**Table 7:** Intervals to the first and second elevations of progesterone (P4), duration of the first luteal phase, intervals to estrus and to pregnancy and interval between lambing in the ewes of each group that began their response to treatments with a normal-length luteal phase

Variable	Treatment1)			

	ME	ME-GnRH	ME-GPG	Total
Days to first P4 elevation	4.2±0.6 (n = 11)	5.0±0.7 (n = 9)	3.1±0.7 (n = 7)	4.2±0.5 (n = 27)
Duration of first luteal phase	10±0.6* (n = 11)	10.4±0.7* (n = 9)	11.4±0.8* (n = 7)	10.5±0.4* (n = 27)
Days to second P4 elevation	21.1±1.2* (n = 11)	19.7±1.4* (n = 9)	19.0±1.9* (n =6)	19.9±0.9* (n = 25)
Days to first estrus	19.9±1.9 (n = 8)	17.2±1.8 (n = 9)	16.0±2.6 (n = 4)	17.7±1.2* (n = 21)
Days to pregnancy	19.9±2.0 (n = 8)	16.9±2.0 (n = 8)	16.0±2.6 (n = 4)	17.9±1.3 (n = 20)
Lambing interval	220.2±3.2 (n = 8)	217.2±2.3 (n = 8)	220.7±3.6 (n = 4)	219.1±1.8 (n = 20)

ME, male effect; GnRH, gonadotrophin releasing hormone; GPG, GnRH-PGF2α-GnRH; PGF2α, prostaglandin F2α.

1) Treatments: ME, ewes were exposed from day 0 to 36 to rams of three different breeds that were rotated every 24 h in groups of 4; ME-GnRH, in addition of exposure to the males the ewes were treated with 4.2 μg of GnRH on day 2; ME-GPG, in addition of exposure to the males the ewes were treated with GnRH on day 2, PGF2α on day 8 and GnRH on day 9.

Differences between groups are not significant for any variable (p>0.05). Values are mean±standard error of the mean. Values in parenthesis indicate the number of animals that contributed to each variable.

* Values with an asterisk are significantly different from the corresponding values for the ewes included in Table 6.
